# Cancer, diabetes, survival and glycemic control: a large multisite analysis

**DOI:** 10.2144/fsoa-2022-0018

**Published:** 2023-01-09

**Authors:** Nina J Karlin, Heidi E Kosiorek, Patricia M Verona, Kyle E Coppola, Curtiss B Cook

**Affiliations:** 1 Division of Hematology & Medical Oncology, Mayo Clinic, Phoenix, Arizona, USA; 2 Information Technology, Mayo Clinic, Phoenix, Arizona, USA; 3 Department of Quantitative Health Sciences, Mayo Clinic, Scottsdale, Arizona, USA; 4 Cancer Registry, Mayo Clinic, Scottsdale, Arizona, USA; 5 Division of Endocrinology, Mayo Clinic, Scottsdale, Arizona, USA

**Keywords:** cancer, oncology, outcomes research

## Abstract

**Aim:**

To determine overall survival (OS) and glycemic control in patients with cancer and diabetes.

**Materials & methods:**

Patients of our institution with breast, colon, lung, pancreas and prostate cancer were retrospectively reviewed. OS was compared between matched patients with and without diabetes, and changes in glucose value over time were assessed.

**Results:**

For 3934 patients each with and without diabetes, adjusted analysis showed no difference in OS according to diabetes status (hazard ratio: 1.07; 95% CI: 0.96–1.20). Mean glucose values decreased over time in patients with and without diabetes (p = 0.01).

**Conclusion:**

In this large study of patients with five common cancers, the co-occurrence of diabetes did not affect OS. Cancer did not adversely affect glucose levels.

## Plain language summary

The aim of this study was to evaluate survival and glucose control in patients with cancer and diabetes at three separate geographic locations in a single health system. From an institutional cancer registry, we identified patients with breast, colon, lung, pancreas and prostate cancers. Patients with and without diabetes were matched by age, sex, cancer type, staging, geographic location and year of cancer diagnosis. In this study, the co-occurrence of diabetes did not affect overall survival. Cancer did not adversely affect glucose levels.

Cancer and diabetes are common diagnoses. Diabetes prevalence continues to increase in the US, and current estimates indicate that nearly 34 million people have the diagnosis [1]. Between 2013 and 2017, an estimated 5,511,635 people alive in the US were diagnosed with cancer [2]. Even though cancer rates are decreasing, the actual number of new cases is increasing because of population growth and aging each year [3]. Moreover, up to 18% of patients with cancer may have coexisting diabetes [4]. As the number of cases of diabetes and cancer increase, it will become more likely that these two conditions will coexist in the same patient. When the two are present in the same patient, care becomes more complex and may require additional specialists to assist with diabetes management and education. Therefore, the mechanism of how diabetes and cancer interact to affect a patient’s outcome is of interest.

One area of study has been the effect of diabetes on survival in patients with cancer. The relationship between cancer and diabetes has been previously examined in a single academic practice, and results showed that diabetes was common in patients with certain solid-organ cancers. In a combined population with different cancer diagnoses, the prevalence of diabetes was 6.8% [5]. The relationship between cancer and diabetes also has been examined in many case–control studies of individual solid and hematologic cancers: breast, lung, prostate, colorectal, pancreatic, gastroesophageal, uterine/ovarian, melanoma, lymphoma, leukemia, squamous cell and neuroendocrine cancers [6–17]. With the exception of melanoma and gastroesophageal and squamous cell cancers, diabetes had no significant effect on death or recurrence in any malignant process studied. Moreover, none of the studies showed adverse effects of cancer on glycemic control 1 year after cancer diagnosis among those with diabetes [6–17].

Data on mortality rates in patients with cancer and coexisting diabetes have been inconsistent. Differences in findings most likely represent variations in methodologic approach to analyses [6,18,19]. Given that the association between cancer, diabetes, survival and glycemic control remains unsettled, ongoing analyses are needed so that healthcare teams can better inform their patients regarding risks. The main limitation of the previous analyses was that data were derived from a single institution (located in the Southwestern US), and the studies often had small sample sizes. However, a convergence of the electronic health record (EHR) across three academic campuses of one institution afforded the opportunity to corroborate the findings on a larger sample size of cancer cases with and without diabetes. In this study, we assessed the interaction between cancer and diabetes from three geographic areas to further examine the questions regarding how diabetes affects cancer survival, and whether a diagnosis of cancer affects glycemic control.

## Materials & methods

### Overview of practice

The cancer center at our institution encompasses academic campuses in the Southeast, Southwest and Midwest (the sites included in this analysis). It is a comprehensive cancer center designated by the National Cancer Institute, with coordinated care and collaboration across the three sites.

### Case selection

Methods similar to those in previous analyses were used for this larger study [6–17], which was approved by our institutional review board. Data on cancer cases from calendar years 2012 through 2018 were retrospectively retrieved from the institutional cancer registry. Cases were identified by pathology results and by *International Classification of Diseases, Ninth Revision* codes. Annual review of the registry is performed, including updated survival information. This analysis was restricted to cancers of the colon, breast, lung, prostate and pancreas to maintain consistency with the previous analysis. Patients younger than 18 years old or who had more than one primary cancer diagnosis were excluded.

This initial cancer dataset was linked to the EHR to identify patients who had a coexisting diagnosis of diabetes (diagnostic code 250.00) corresponding around the time of cancer diagnosis. Data on hemoglobin A_1c_ (HbA_1c_) and glucose levels during the first 12 months after cancer diagnosis were extracted. The glucose detection method uses hexokinase and is the same at all sites [20]. Patients with diabetes (cases) were matched 1:1 using a greedy algorithm to patients with cancer but no diabetes (controls) to yield the final analytic dataset. Variables included in the matching algorithm were cancer type, stage, geographic location of care, age, sex and year of cancer diagnosis [6–17]. EHR claims during the preceding and concurrent year from the date of cancer diagnosis were used to calculate the Charlson comorbidity index (CCI) [21]. The CCI was modified to exclude diabetes from the calculation to capture the prevalence of comorbid conditions other than diabetes.

Previous studies have shown no significant association of diabetes related therapies with cancer outcomes, so these were not included in this analysis [7–13]. In addition, cancer therapies are varied (e.g., cytotoxic chemotherapy, targeted therapies, immunotherapies, radiation and surgery) and also vary by cancer type. Moreover, cancer therapeutic protocols are standardized across all three sites, and little difference would be expected in outcomes on the basis of treatment. Therefore, these were not reviewed.

### Statistical analyses

Statistical analyses were similar to those described in prior reports [6–17]. Demographic and clinical variables were compared between matched groups with and without diabetes by use of paired t*-*tests for continuous variables and McNemar tests for categorical variables. For comparisons between geographic locations, χ^2^ tests or analysis of variance were used. Overall survival (OS) was calculated from time of cancer diagnosis until death. OS was estimated with the Kaplan–Meier method, and survival curves were compared between diabetes and non diabetes groups by use of the log-rank test for overall and cancer types separately. Cox proportional hazards models were used to assess OS and included matched pairs as a strata variable. An additional Cox model was constructed that included CCI (without diabetes) as a covariate. Linear mixed models evaluated HbA_1c_ levels during the first year after cancer diagnosis for patients with diabetes only, because values were unavailable for most patients without diabetes. A similar approach was used for modeling glucose values during the same time frame in both groups. Fixed effects included time since cancer diagnosis (days), designation of case or control, an interaction term (days × case–control designation) and patient-specific random effects. SAS version 9.4 (SAS Institute Inc.) was used for analysis, and p-value less than 0.05 were considered statistically significant. Data were analyzed and reported in aggregate (overall for all cancers and all locations combined).

## Results

### Patient characteristics

A total of 7868 patients were analyzed from all the three geographic locations (3934 with diabetes, matched with 3934 without diabetes) (Table 1). The mean (SD) age was 67 (10) years at diagnosis. The majority of patients (60.0%) were from the Midwest location, with 21.5% from the Southeast and 18.6% from the Southwest location. A greater proportion of patients with diabetes were of race other than White (12.5 vs 7.9%; p < 0.001), and patients with diabetes had a significantly higher CCI score (6.2 vs 5.4; p < 0.001). Prostate cancer was the most common cancer type (n = 2656; 33.8%), followed by lung (n = 1794; 22.8%), breast (n = 1422; 18.1%), pancreas (n = 1344; 17.1%) and colon cancer (n = 652; 8.3%). Race, CCI, and age at cancer diagnosis differed significantly across geographic locations (Table 2).

**Table 1.** Patient demographics by diabetes status for total cohort.

Characteristic	Group^†^		p-value
	Diabetes (n = 3934)	No diabetes (n = 3934)	
Age at cancer diagnosis, years	67 (10)	67 (10)	NA

Sex			NA
Women	1467 (37.3)	1467 (37.3)	
Men	2467 (62.7)	2467 (62.7)	

Site			NA
Southwest	730 (18.6)	730 (18.6)	
Southeast	844 (21.5)	844 (21.5)	
Midwest	2360 (60.0)	2360 (60.0)	

Race			<0.001^‡^
White	3443 (87.5)	3625 (92.1)	
Other than White	491 (12.5)	309 (7.9)	

TNM staging			NA
1	1095 (27.8)	1095 (27.8)	
2	1421 (36.1)	1421 (36.1)	
3	594 (15.1)	594 (15.1)	
4	824 (20.9)	824 (20.9)	
Weighted CCI	6.2 (3.7)	5.4 (3.5)	<0.001^§^

Year of cancer diagnosis			NA
2012	608 (15.5)	596 (15.1)	
2013	575 (14.6)	599 (15.2)	
2014	593 (15.1)	581 (14.8)	
2015	640 (16.3)	654 (16.6)	
2016	657 (16.7)	648 (16.5)	
2017	682 (17.3)	699 (17.8)	
2018	179 (4.5)	157 (4.0)	

† Values are mean (SD) or no of patients (%).

‡ χ^2^ test.

§ Paired t-test.

CCI: Charlson comorbidity index; NA: Not applicable, matched variable.

**Table 2.** Patient demographics by geographic location for total cohort.

Characteristic	Location^†^			p-value
	Southwest (n = 1460)	Southeast (n = 1688)	Midwest (n = 4720)	
Age at cancer diagnosis, years	68 (9)	67 (9)	67 (10)	<0.001^‡^

Sex				NA
Women	444 (30.4)	744 (44.1)	1746 (37.0)	
Men	1016 (69.6)	944 (55.9)	2974 (63.0)	

Cancer site				NA
Breast	286 (19.6)	374 (22.2)	762 (16.1)	
Colon	80 (5.5)	110 (6.5)	462 (9.8)	
Lung	264 (18.1)	402 (23.8)	1128 (23.9)	
Pancreas	154 (10.5)	294 (17.4)	896 (19.0)	
Prostate	676 (46.3)	508 (30.1)	1472 (31.2)	

Diabetes				NA
No	730 (50.0)	844 (50.0)	2360 (50.0)	
Yes	730 (50.0)	844 (50.0)	2360 (50.0)	

Race				<0.001^§^
White	1305 (89.4)	1393 (82.5)	4370 (92.6)	
Other than White	155 (10.6)	295 (17.5)	350 (7.4)	

TNM staging				NA
1	424 (29.0)	494 (29.3)	1272 (26.9)	
2	482 (33.0)	598 (35.4)	1762 (37.3)	
3	272 (18.6)	234 (13.9)	682 (14.4)	
4	282 (19.3)	362 (21.4)	1004 (21.3)	
Weighted CCI	5.1 (3.5)	5.5 (3.5)	6.1 (3.7)	<0.001^‡^

Cancer diagnosis, year				NA
2012	192 (13.2)	234 (13.9)	778 (16.5)	
2013	208 (14.2)	218 (12.9)	748 (15.8)	
2014	221 (15.1)	233 (13.8)	720 (15.3)	
2015	245 (16.8)	255 (15.1)	794 (16.8)	
2016	245 (16.8)	267 (15.8)	793 (16.8)	
2017	283 (19.4)	300 (17.8)	798 (16.9)	
2018	66 (4.5)	181 (10.7)	89 (1.9)	

† Values are mean (SD) or no of patients (%).

‡ Analysis of variance F test.

§ χ^2^ test.

CCI: Charlson comorbidity index; NA: Not applicable, matched variable.

### Cancer survival

In unadjusted analyses, after a median follow-up of 36.8 months, and when including all cancers aggregated by type and geographic location, patients with diabetes had significantly lower OS than patients without diabetes (hazard ratio: 1.16; 95% CI: 1.04–1.29; p = 0.007) (Table 3). A similar finding was noted for breast cancer (hazard ratio: 1.62; 95% CI: 1.09–2.41; p = 0.02). After adjusting for CCI; however, these comparisons were no longer significant. In addition, no differences in OS between patients with and without diabetes were noted for colon, lung, pancreatic and prostate cancers (Table 3). Analysis of OS according to geographic location showed no significant differences after adjusting for age, cancer type, race, sex, cancer stage and CCI (data not shown).

**Table 3.** Unadjusted and adjusted OS for patients with cancer and coexisting diabetes (compared with no diabetes), total cohort.

Cancer type	Unadjusted OS		Adjusted OS	
	HR (95% CI)	p-value	HR (95% CI)	p-value
All	1.16 (1.04–1.29)	.007	1.07 (0.96–1.19)	0.23
Breast	1.62 (1.09–2.41)	.02	1.39 (0.91–2.13)	0.13
Colon	1.21 (0.86–1.69)	.27	1.10 (0.78–1.56)	0.59
Lung	1.14 (0.96–1.34)	.13	1.04 (0.88–1.24)	0.64
Pancreas	1.11 (0.93–1.34)	.26	1.08 (0.90–1.31)	0.41
Prostate	1.05 (0.74–1.50)	.79	0.81 (0.54–1.22)	0.31

HR: Hazard ratio; OS: Overall survival.

### Glycemic control

**Figure 1.**
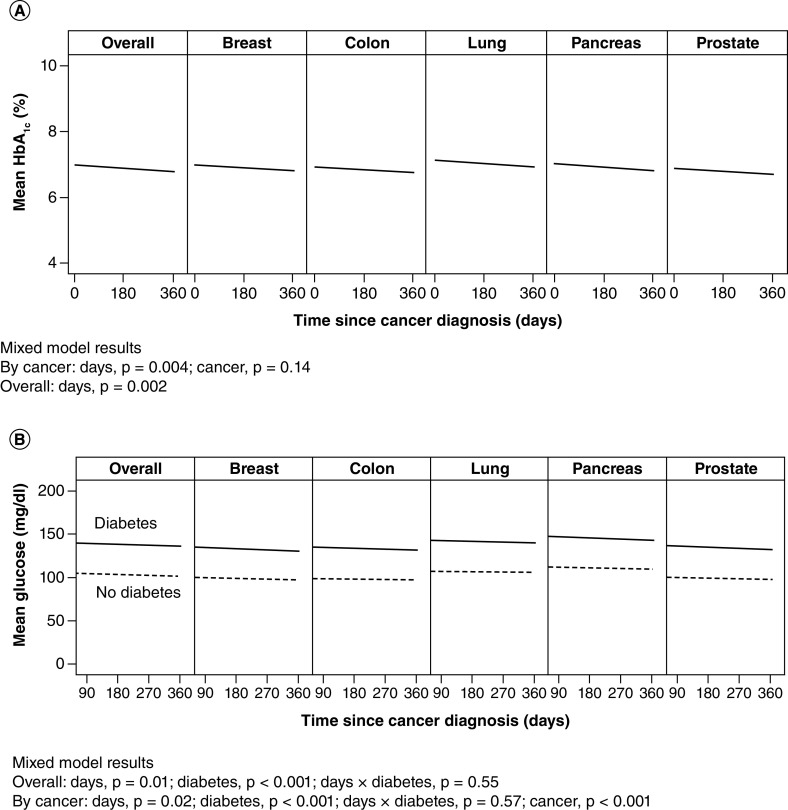
Glucose control for 1 year after cancer diagnosis. Data overall (all cancers, all sites) and for individual cancers (aggregate all sites). **(A)** Hemoglobin A_1c_ (HbA_1c_) values for patients with diabetes only. **(B)** Glucose values for patients with and without diabetes.

Among patients with diabetes, 2049 (52.1% overall; 60% breast, 55% colon, 37% lung, 56% pancreas, 56% prostate cancer) had at least 1 HbA_1c_ measurement during the year after cancer diagnosis; the overall mean (SD) HbA_1c_ value was 6.9% (1.3%). Over a 1-year follow-up period, the overall HbA_1c_ significantly decreased (p = 0.002) (Figure 1A). HbA_1c_ decreased significantly in all separate cancer diagnoses (p = 0.004), but no group differences were identified between cancer type (p = 0.14).

Among patients with diabetes, 1859 (47.3%) had at least one glucose measurement during the year after cancer diagnosis; mean (SD) value was 139 (51) mg/dl. In patients without diabetes, 1656 (42.1%) had at least one glucose measurement; mean (SD) value for these patients was 103 (18) mg/dl, which was significantly lower than in the diabetes group (p < 0.001) (Figure 1B). Overall, glucose value significantly decreased over time among those with diabetes (p = 0.01). When cancer type was included in the mixed model, observations were similar: a decrease in glucose value over time among diabetes patients (p = 0.02). In addition, mean glucose value differed according to cancer type (p < 0.001) and was highest among those with pancreatic and lung cancers (Figure 1B).

## Discussion

It is still not clear how diabetes and solid-organ cancers interact to affect one another’s outcome. For example, data on survival have been inconsistent, with some studies showing that coexisting diabetes was associated with decreased survival and others not showing this outcome [6,18,19]. A series of case–control analyses, comparing patients with and without diabetes, previously examined site-specific outcomes among patients with newly diagnosed breast, lung, colorectal, pancreas and prostate cancers. With few exceptions (e.g., in gastroesophageal cancer, squamous cell cancers of the head and neck and melanoma), these studies showed that coexisting diabetes was not associated with an increased mortality rate [6–17]. This observation was consistent regardless of the data source – whether from institutional mortality data or the National Death Index [22,23]. In addition, cancer did not affect glycemic control during a 1-year follow-up period in patients with or without diabetes [6–17].

The convergence of our EHR platform presented an opportunity to expand the previous analyses to a larger sample size and to assess differences across the geographic campuses of one healthcare system. Some differences were noted between diabetes and non diabetes patients (race and CCI) and across geographic locations (age at cancer diagnosis, race and CCI), although these are largely due to the differences in distribution of cancer types seen at the various locations. For instance, the Southwest site included a larger proportion of patients with prostate cancer than the other two sites. However, in adjusted analyses, no differences in OS were noted between patients with and without diabetes overall or for individual cancer types. Furthermore, no differences were seen in OS between geographic locations. Results of this study corroborate previous single-site findings. At least in this patient population, findings can be reassuring to patients and their healthcare team that the presence of diabetes does not decrease survival.

Several factors may affect glycemic control among patients with diabetes during treatment of cancer. For instance, glucocorticoids, which are often used during chemotherapy, could lead to acute exacerbations of hyperglycemia in patients with and without diabetes. In contrast, weight loss due to decreased appetite could result in decreased glucose levels. Results of analysis of this larger dataset support those of earlier studies indicating that glycemic control does not worsen during the first year after cancer diagnosis.

It is reassuring that OS and glycemic outcomes did not differ according to geographic location of care. Differences in demographics and comorbid conditions across sites could lead to variations in outcome. However, convergence of our EHR also brought a merging of treatment protocols across the healthcare system. These standardized approaches to care could mitigate any influence of differences in other factors on the outcomes examined here.

The results of this study confirm those of previous studies that diabetes does not affect survival in breast, colon, lung, pancreatic and prostate cancers, but similar observations have not been true in other cancer types. For instance, diabetes was associated with decreased OS in gastroesophageal and squamous cell cancers and with shorter progression-free survival in melanoma [11,12,16]. The methods of the current study; however, could be used to reexamine these relationships on a larger and broader geographic scale.

Strengths of this study include the large sample size, inclusion of data from three geographic locations and results controlled for comorbid conditions. Nonetheless, there were several limitations. Although derived from three different geographic areas of care, the data were still from within the same healthcare system, in which cancer treatment protocols have been converged and standardized. It may not be unexpected, therefore, that outcomes would be similar. In addition, the affected population was mostly White. Different results may be obtained when examining OS and glycemic control from healthcare systems with populations that are more ethnically diverse or of lower socioeconomic status, which was not assessed in this analysis.

## Conclusion

Results of this three-site study confirmed previous single institution results that diabetes does not affect OS and that cancer does not affect glycemic control in a population of patients with breast, colon, lung, pancreas and prostate cancers. Expanded analyses are needed from other healthcare facilities to determine whether these findings are common or unique to one healthcare system.

## Future perspective

These findings should reassure providers that diabetes does not affect OS of patients with breast, colon, lung, pancreas and prostate cancers. Furthermore, cancer does not affect glycemic control in patients with diabetes.

**Summary points**

- The number of patients with coexisting cancer and diabetes will most likely increase.
- Information on the interaction between cancer, diabetes and survival is needed.
- In this large study of patients with five cancer types, diabetes did not affect survival.
- A diagnosis of cancer did not adversely affect glycemic control.
- Continued study from other health facilities is needed to determine whether these findings are common or unique to one healthcare system.
- Continued study is needed to confirm findings in more diverse population groups.
- Analyses can be extended to hematologic cancers to assess for similar findings regarding diabetes and survival in these populations.
- At least in the patient populations analyzed here, findings can be reassuring to patients and their healthcare teams that the presence of diabetes does not decrease cancer survival.
